# van der Waals devices for surface-sensitive experiments

**DOI:** 10.1039/d5nr02125a

**Published:** 2025-08-14

**Authors:** Nicolai Taufertshöfer, Corinna Burri, Rok Venturini, Iason Giannopoulos, Sandy Adhitia Ekahana, Enrico Della Valle, Anže Mraz, Yevhenii Vaskivskyi, Jan Lipič, Alexei Barinov, Dimitrios Kazazis, Yasin Ekinci, Dragan Mihailovic, Simon Gerber

**Affiliations:** a PSI Center for Photon Science, Paul Scherrer Institute 5232 Villigen PSI Switzerland simon.gerber@psi.ch; b Institute for Quantum Electronics, ETH Zurich 8093 Zurich Switzerland; c Laboratory for Solid State Physics and Quantum Center, ETH Zurich 8093 Zurich Switzerland; d Department of Complex Matter, Jožef Stefan Institute 1000 Ljubljana Slovenia dragan.mihailovic@ijs.si; e CENN Nanocenter 1000 Ljubljana Slovenia; f Elettra – Sincrotrone Trieste 34149 S.C.p.A. Basovizza (TS) Italy; g Faculty of Mathematics and Physics, University of Ljubljana 1000 Ljubljana Slovenia

## Abstract

In-operando characterization of van der Waals (vdW) devices using surface-sensitive methods provides critical insights into phase transitions and correlated electronic states. Yet, integrating vdW materials in functional devices while maintaining pristine surfaces is a key challenge for combined transport and surface-sensitive experiments. Conventional lithographic techniques introduce surface contamination, limiting the applicability of state-of-the-art spectroscopic probes. We present a stencil lithography-based approach for fabricating vdW devices, producing micron-scale electrical contacts, and exfoliation in ultra-high vacuum. The resist-free patterning method utilizes a shadow mask to define electrical contacts and yields thin flakes down to the single-layer regime *via* gold-assisted exfoliation. As a demonstration, we fabricate devices from 1T-TaS_2_ flakes, achieving reliable contacts for application of electrical pulses and resistance measurements, as well as clean surfaces allowing for angle-resolved photoemission spectroscopy. The approach provides a platform for studying the electronic properties of vdW systems with surface-sensitive probes in well-defined device geometries.

## Abstract 2

We present a fabrication process that combines contact patterning with a stencil and gold-assisted exfoliation of van der Waals materials, producing pristine surfaces and micron-scale device geometries on thin flakes down to the monolayer limit.

## Graphical abstract

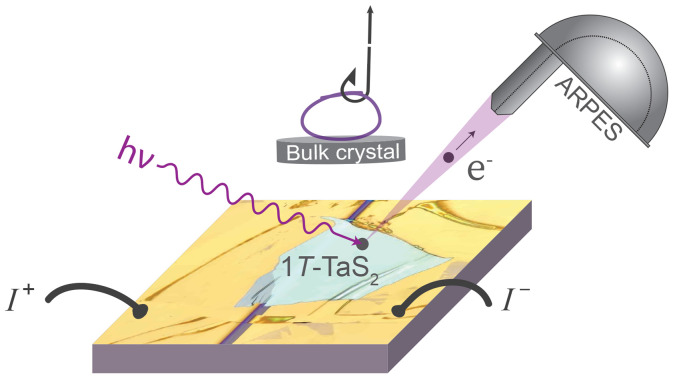

## 1 Introduction

van der Waals (vdW) materials provide a rich playground for exploring correlated electronic states and phase transitions in two-dimensional systems, with applications in next-generation electronic devices based on thin vdW flakes. They enable the exploration of novel quantum states with unprecedented control *via* gating and pulse application.^1–5^ While transport measurements provide insight into the electronic properties of such devices, spectroscopic tools are necessary to directly probe the electronic band structure. Combining these approaches remains challenging as conventional lithographic device fabrication methods introduce surface contamination or modify the surface, preventing the use of state-of-the-art surface-sensitive tools, such as scanning tunneling microscopy^6^ or angle-resolved photoemission spectroscopy (ARPES).^7^ Thus, a critical challenge in fabricating devices for surface-sensitive experiments involving vdW materials is achieving three aspects simultaneously: (i) obtaining thin, uniform flakes down to monolayers, (ii) integrating electrical contacts at the micron-scale for well-defined device geometries, and (iii) maintaining pristine surface quality through *in situ* exfoliation in ultra-high vacuum (UHV).

A variety of fabrication methods have been developed to integrate vdW materials into devices, but most approaches satisfy only two out of the three requirements above (see Table 1). Fabrication methods involving standard dry pick-up transfer techniques with viscoelastic polymer stamps, *e.g.* polydimethylsiloxane (PDMS),^8^ are widely used for assembling vdW heterostructures, but they leave polymer residues and water on the surface, which degrade the interface quality and limit compatibility with surface-sensitive techniques.^9,10^ While such contamination can in principle be removed, *e.g.* from graphene devices by annealing at ≈350 °C,^11^ this approach is viable only for certain “robust” vdW materials. In many cases, such treatment leads to irreversible structural changes. For instance, for the prototypical transition metal dichalcogenide material 1T-TaS_2_, annealing at ≈325 °C induces a polytype transition to the semimetallic 2H phase.^12^ Alternative strategies to avoid polymer contamination include protective capping with hBN or graphene^13^ during transfer or the use of metalized SiN_*x*_ membranes.^14^ Though, both approaches still require mild annealing and are difficult to integrate with patterned device architectures.

**Table 1:** Comparison of fabrication methods for vdW devices in terms of: (i) monolayer/thin flake preparation, (ii) micron-scale contact integration, and (iii) pristine surface preservation

Method	(i)	(ii)	(iii)
Dry pick-up transfer^8,11,13,14^	✓	✓	×^a^
UHV cleaving of bulk crystals with pre-attached contacts^15,16^	×	×	✓
Glovebox device assembly^17–20^	✓	✓	×^a^
UHV fabrication system^21^	✓	×	✓
Gold-assisted exfoliation^22–25^	✓	×^b^	✓
Stencil + gold-assisted exfoliation (this work)	✓	✓	✓

a Clean surfaces can be achieved, but only by annealing.

b Patterning is possible, but it compromises the surface cleanliness.

Cleaving bulk vdW crystals in UHV with pre-attached contacts has recently also been used in transport-ARPES studies on Ca_2_RuO_4_ ^15^ and 1T-TaS_2_.^16^ This method exposes a pristine surface *in situ* but is limited to bulk materials, making it incompatible with micron-scale device designs.

Fabrication in inert atmospheres such as in N_2_ or Ar gloveboxes,^17–20^ or even under UHV,^21^ has been explored to exfoliate thin flakes and assemble heterostructures while minimizing oxidation. However, even within a controlled N_2_ environment, water adsorption on the surface remains an issue, often necessitating an annealing step to obtain clean surfaces. Moreover, such fabrication setups require specialized equipment that is rarely available near UHV measurement facilities.

Finally, gold-assisted exfoliation^22–25^ allows for preparation of large-area monolayer flakes with clean surfaces by leveraging the strong interaction between freshly evaporated Au and chalcogen atoms. While Au is most commonly used due to its inert nature, it can be replaced by other metals such as Pd, Ni, Cu, and Ag, which exhibit similarly high adhesion through strong bonding to chalcogen atoms.^26^ Importantly, the timing of exfoliation is critical: contaminants adsorb on Au within minutes, making the surface hydrophobic, which significantly reduces the monolayer yield.^24,27^ This degradation cannot be reversed by cleaning or annealing, precluding post-processing steps such as metal lift-off. While the method yields high-quality flakes, device integration typically involves patterning steps after exfoliation, for example, by etching in potassium iodide solution.^25^

**Fig. 1:**
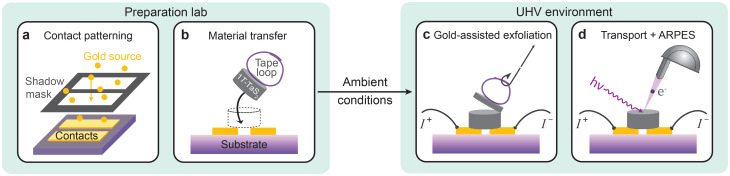
Schematic of the vdW device fabrication process using a stencil for contact patterning and gold-assisted exfoliation. (a) Au evaporation through a shadow mask, defining the contact geometry. (b) Bulk vdW material on a tape loop is transferred to the pre-patterned contacts immediately after Au evaporation. (c) Gold-assisted exfoliation in UHV. (d) Integration of surface-sensitive techniques, *e.g.* ARPES, and *in situ* transport measurements.

To fulfill all three criteria (i)–(iii) simultaneously (see Table 1), we introduce a fabrication method that utilizes gold-assisted exfoliation with pre-patterned metallic contacts defined by evaporation through a stencil. This approach enables the fabrication of vdW devices with micron-scale contact geometries while preserving pristine surfaces through exfoliation in UHV. Unlike approaches that require integrated lithography clusters, our method allows for device fabrication in a standard laboratory environment and transport to the experimental facility, *e.g.* synchrotrons or other dedicated UHV measurement setups, without specialized vacuum equipment. The fabrication process, illustrated in Fig. 1, consists of three main steps: first, Au contacts are deposited on a Si/SiO_2_ substrate through a shadow mask, defining the device geometry. Second, immediately after venting the evaporation chamber, a freshly cleaved bulk crystal is transferred to the contacts using a tape loop. The device is then assembled on a sample holder and can be transported under ambient conditions. Third, exfoliation is performed inside the UHV measurement chamber by removing the tape, which cleaves the bulk crystal and exposes clean vdW flakes over the Au contacts.

As a demonstration of this technique, we fabricate two-terminal devices of 1T-TaS_2_, a prototypical vdW material due to its rich electronic phase diagram, including a Mott insulating low-temperature state,^28–32^ different charge-density wave (CDW) phases,^33,34^ superconductivity upon pressure,^35^ and a putative quantum spin liquid state.^36,37^ Furthermore, 1T-TaS_2_ exhibits a non-equilibrium metallic “hidden” state that can be reversibly induced by optical or electrical pulses.^4,38–41^ This non-volatile state holds promise for use in the next generation of cryo-memory devices due to energy efficiency and switching speed.^42–44^

The devices presented here are tailored for micro-beam ARPES^45,46^ in combination with transport measurements and application of electrical (switching) pulses. More broadly, the fabrication method enables studies of the electronic properties of thin vdW flakes and provides a platform for integrating surface-sensitive techniques with transport measurements across a wide range of material systems.

## 2 Results and discussion

### 2.1 Device fabrication

**Fig. 2:**
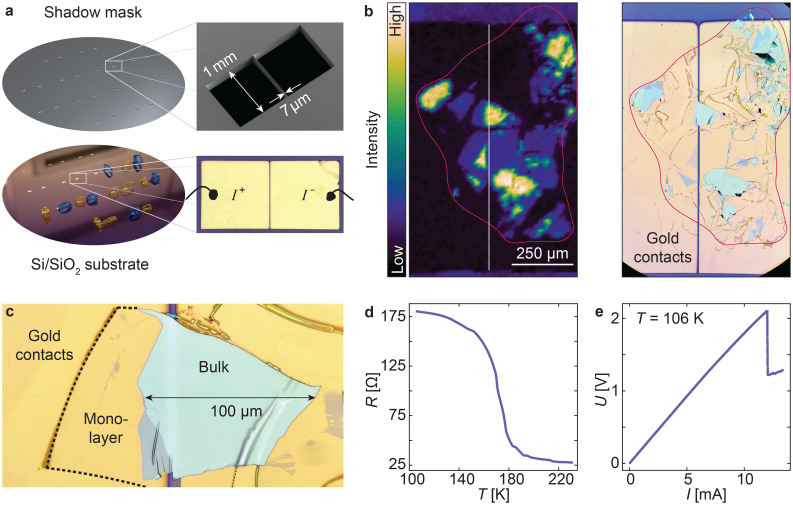
Fabrication and characterization of a two-terminal 1T-TaS_2_ device. (a) Shadow mask with 24 copies of the device geometry (top) used to pattern a 4-inch Si/SiO_2_ wafer with Au evaporation (bottom), on which tape loops with bulk 1T-TaS_2_ crystals are then placed. (b) Ta 4f core-level intensity map from spatially-resolved ARPES alongside an optical microscope image of the cleaved 1T-TaS_2_ flakes. The outline of the bulk crystal is shown in red. (c) Optical microscope image of a 45 nm thick bulk flake bridging the gap between the contact pads, as well as an extended monolayer flake. (d) *In situ* transport measurements of the 1T-TaS_2_ device, showing the metal–insulator (NCCDW-CCDW) transition upon cooling. (e) Pulsed *IV* measurements in the insulating CCDW state show electrical switching to the “hidden” state, marked by a voltage drop at 12 mA.

Here, we outline the steps for the preparation of the vdW device. Details of the shadow-mask fabrication are provided in the Methods section. To benefit from the high adhesion of gold-assisted exfoliation, it is crucial to fabricate electrodes in a way that avoids contamination. This can be achieved through evaporation of Au through a shadow mask.^47–50^ Such apertures can be made using various techniques, such as mechanical cutting, focused ion beam milling,^51–53^ or etching into Si wafers,^54–56^ with the choice depending on the required feature size. For our approach, we employ laser lithography combined with deep reactive ion etching (DRIE)^57^ to produce high-aspect ratio masks with well-defined micron-scale features (see Fig. 2a and the Methods). This stencil wafer is placed on the Si/SiO_2_ substrate during the deposition of Ti/Au contacts *via* electron beam evaporation. After the contact deposition, bulk 1T-TaS_2_ crystals with typical dimensions of 1–3 mm in diameter are cleaved on tape loops, positioned over the gap between the Au pads, brought into contact with the substrate, and gently pressed to ensure adhesion.

Due to rapid degradation of the Au surface in air, it is crucial that this is performed within ≈5 min after venting the evaporation chamber to obtain a high yield of thin flakes.^24^ Next, electrical connections are made by attaching wires to the contact pads using Ag epoxy. A fully assembled device is shown in Fig. S1.

To obtain a contamination-free surface, the exfoliation process is performed later under UHV conditions by removing the tape loop with a mechanical manipulator. This process cleaves the vdW crystal, leaving flakes with freshly exposed surfaces on the contacts at random positions; only flakes that bridge the gap between the contact pads form functional devices. Electrical connectivity is tested and the flakes are visually inspected using an optical microscope, as well as in our ARPES demonstration the spectral intensity of the Ta 4f core levels, mainly to confirm flake dimensions, thickness, and surface homogeneity (see Fig. 2b). The vdW flakes, like the one shown in Fig. 2c, have lateral dimensions from tens to hundreds of microns. Atomic force microscopy (AFM) reveals thicknesses ranging from a monolayer up to ≈100 nm (see Fig. S2), enabling access to both few-layer and bulk properties. Such variation in thickness can occur even within a single macroscopic flake as local tearing and a non-uniform stress distribution during peeling, combined with the material's in-plane stiffness, can result in different exfoliation planes. In our experiments, we routinely observe mono-, bi-, and few-layer flakes. While the exfoliation process is inherently non-deterministic, the yield and thickness distribution of flakes can be influenced by tuning the Au surface properties—such as roughness and cleanliness—through parameters like evaporation rate, film thickness, adhesion layer, and time elapsed after breaking vacuum. This enables the selection of flakes with the desired thickness from the exfoliated set.

### 2.2 Device characterization

#### Transport measurements

Fig. 2d shows the temperature dependence of the two-terminal resistance upon cooling after exfoliation at 250 K, including the metal–insulator transition from the nearly-commensurate (NC) to commensurate (C) CDW phase of 1T-TaS_2_. Large contact resistances on the order of several hundreds of Ω are typically observed when vdW materials are probed with metal contacts on their flat face.^10^ However, we observe exceptionally low contact resistances on the order of 10 Ω in all our devices, *e.g.* the two-terminal resistance of the device shown in Fig. 2c is 25 Ω at room temperature, which we attribute to the large contact area with covalent-like bonding at the 1T-TaS_2_/Au interface.^25^ The high-quality contacts allow for low-voltage state switching of the 1T-TaS_2_ device, where electrical excitation induces a phase transition from the insulating, equilibrium CCDW phase to a metallic, non-thermal and metastable “hidden” state.^38^ This phase transition is characterized by a drop in resistance and associated with the disruption of long-range CCDW order. Recent studies suggest that the emergence of the hidden state is driven by out-of-plane restacking and the disappearance of interlayer dimerization of the CCDW phase.^32,39–41^ In our case, at *T* = 106 K, we observe a resistance drop from 186 to 96 Ω upon application of a 2.1 V, 200 μs voltage pulse (see Fig. 2e). Although the switching to the hidden state is apparent from the transport measurements, the short lifetime of this metastable phase at *T* > 50 K prevents direct detection by techniques with longer acquisition times, such as ARPES.^42^

#### Angle-resolved photoemission spectroscopy

**Fig. 3:**
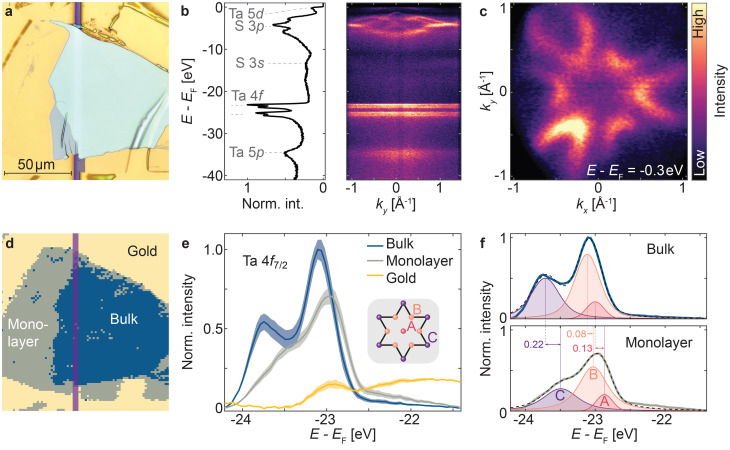
Spatially-resolved ARPES of a 1T-TaS_2_ device. (a) Optical microscope image of the measured flake. (b) ARPES spectrum with the angle-integrated elemental signatures (left) and the corresponding band structure (right). (c) Isoenergy contour integrated over a 0.1 eV window centered at *E* − *E*_F_ = −0.3 eV. Streaks near normal emission in *k*_*y*_ in panels b and c are artifacts from the analyzer's deflector system. (d) *k*-means clustering map (the spatial extent corresponds to panel a), identifying three distinct regions based on the Ta 4f core-level spectrum. The contact gap is marked in purple. (e) Averaged Ta 4f_7/2_ core-level spectrum for all data points within the clusters. The shaded area corresponds to the respective standard deviation. The inset depicts the star-of-David structure of Ta atoms which forms the building block of the CDW states of 1T-TaS_2_ and features three distinct Ta species: the central site A, as well as the inner and outer ring site B and C, respectively. (f) Fits of the Ta 4f_7/2_ bulk and monolayer spectra using three Voigt profiles, corresponding to the three inequivalent Ta environments. The sum of the three fitted components is depicted by dashed lines, whilst the experimental data is shown in bold.

Fig. 3 summarizes spatially-resolved ARPES measurements performed on the device shown in Fig. 2, using a photon energy of 74 eV and a beam spot focused to 1.0 × 1.5 μm^2^. Fig. 3b shows representative angle-integrated and momentum-resolved spectra of this flake. In particular, the dispersion of the main valence bands, formed by S 3p orbitals,^34^ is well resolved (see also Fig. S3). This is further validated by a constant energy contour at *E* − *E*_F_ = −0.3 eV with details and resolution comparable to those in studies on bulk crystals.^40,58–61^

A spatially resolved Ta 4f core-level scan with a step size of 1.5 μm is used to group the data points based on spectral similarity by the *k*-means clustering algorithm^62^ (see Fig. 3d). This unsupervised learning algorithm partitions the dataset into *k* clusters by minimizing the intra-cluster variance. We determine the optimal *k* using the “elbow” method, where diminishing returns in reducing the within-cluster sum of squares are observed. We obtain *k* = 3 (see Fig. S4), where the three clusters correspond to distinct regions of the scanned area: bulk (>30 layers) and monolayer 1T-TaS_2_ flake, as well as Au surface, agreeing well with the optical microscope image (see Fig. 3a and d, as well as the overlaid image in Fig. S5). All angle-integrated spectra within a cluster are averaged to obtain the mean and standard deviation shown in Fig. 3e. A Shirley-type background^63^ is subtracted to account for inelastic scattering (see Fig. S6).

Within the bulk-like cluster, the Ta 4f core levels exhibit a splitting stemming from the different electronic environments of Ta atoms within the star-of-David-shaped lattice distortion in the CDW phase.^33^ As shown in the inset in Fig. 3e, there are three distinct Ta sites, namely a central (A), as well as an inner (B) and outer (C) ring position. Due to their different electronic environment and the charge redistribution towards the central A site, the Ta 4f_7/2_ core levels appear at different binding energies, *i.e. E* − *E*_F_ = −23.7 eV (C), −23.1 eV (B), and −23.0 eV (A) in accordance with measurements of bulk single-crystals.^64^ The ratio of the integrated intensities matches that of the Ta atoms of each species, *i.e.* A : B : C ≙ 1 : 6 : 6. We attribute deviations of the fit from the experimental data, most pronounced for the C species, to inequivalent C sites due to the complex out-of-plane stacking,^31,32^ or puckering distortions^65^ present in the CDW states of 1T-TaS_2_. The discrepancy at the edge of the spectrum can be attributed to systematic errors from the background subtraction.

In contrast, the spectrum taken from the monolayer cluster shows a reduction of the splitting between the B and C species, alongside a reduced relative C intensity, which we attribute to the absence of out-of-plane stacking. A shift of ≈0.1 eV towards lower binding energies is observed, which we ascribe to charge transfer from Au to 1T-TaS_2_ and is consistent with findings in monolayer MoS_2_ on Au contacts.^24^ To study the influence of 1T-TaS_2_/Au interface in more detail, one could aim for a device with a 1T-TaS_2_ monolayer that extends over the gapped region of the substrate in a future experiment, allowing for a direct comparison of the Au-bound and freestanding monolayer.

## 3 Conclusions

We have developed a resist-free device fabrication method tailored for exfoliation of vdW materials, combining contact patterning through a stencil with gold-assisted exfoliation under UHV conditions. This approach enables well-defined device geometries with micron-sized features, including electrical connections for transport measurements and switching. The ability to fabricate devices under ambient conditions while performing exfoliation in UHV makes the approach particularly suitable for use with surface-sensitive techniques. As a proof of principle, we fabricate two-terminal devices from 1T-TaS_2_ flakes, showcasing the feasibility of simultaneous electrical and ARPES measurements. We find that electrical contacts to the flakes are of high quality with contact resistances of only ∼10 Ω. Spatially-resolved micro-beam ARPES demonstrates that the our device fabrication yields high-quality surfaces across ∼100 μm sized flakes with spectroscopic detail comparable to traditional measurements on bulk crystals. Our work presents a platform for studying devices made from vdW materials down to the single-layer regime and enables combined transport and spectroscopic investigations that so far have not been permitted by other fabrication methods.

## 4 Experimental methods

### 4.1 Material synthesis

Single crystals of 1T-TaS_2_ are grown by chemical vapor transport using I_2_ as a transport agent, following previously established procedures.^4^

### 4.2 Shadow mask fabrication

Stencils are fabricated from 250 μm thick Si wafers utilizing laser lithography and DRIE. The electrode design is exposed on a 9 μm thick film of positive tone photoresist (SPR 220 7.0, micro resist technology) using a direct-write laser lithography tool (DWL 66+, Heidelberg Instruments Mikrotechnik) at 405 nm. The Si wafer is etched through in a DRIE tool (Omega Rapier 200 mm process module, SPTS Technologies) using a variation of the highly anisotropic “Bosch process” optimized for low sidewall roughness and micron-scale features. It consists of alternating etching and passivation cycles. The combination of physical and chemical mechanisms enables highly directional etching through the entire wafer, while minimizing lateral etching and ensuring vertical sidewalls.

**Fig. 4:**
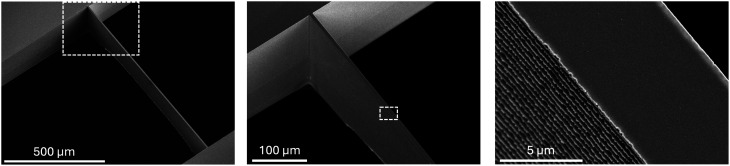
Scanning electron microscopy images of the shadow mask with a 7 μm wide, 1 mm long Si bridge at different magnifications (enlargements of the dashed boxes are shown on the right). The image on the right reveals the steep sidewalls with a characteristic scalloped profile, arising from the alternating passivation and etching cycles during the Bosch DRIE process.

Our process is carried out at 0 °C with the electrostatic chuck biased at 6000 V and the coil current set to 10 A. The sequence of three steps (see the process parameters listed in Table 2) starts with Si etching from the reaction between F radicals, from SF_6_ plasma, and Si that results in volatile SiF_4_ byproducts. Additional platen power is applied to accelerate plasma-generated ions toward the surface and remove material selectively. The chemical nature of this process leads to unwanted lateral etching. To counteract this effect, our process incorporates a passivation step in which isotropic C_4_F_8_ plasma deposits a fluorocarbon polymer layer on both the surface and the sidewalls of the etched trenches. This is followed by an intermediate base etching step using a gaseous mixture of SF_6_ and O_2_. Platen bias power is applied to enhance anisotropy and remove most of the passivation layer at the base of the trenches, while preserving it on the sidewalls to protect them during the subsequent Si etch which continues until the passivation layer is completely consumed. These steps are repeated cyclically, where each cycle (see Fig. 4) removes a controlled amount of Si and prevents lateral etching, enabling the formation of high-aspect-ratio structures.

**Table 2:** Parameters of the Bosch DRIE process

Process step	Passivation	Base etch	Si etch
Etch time [s]	1.8	1.1	4.0
Pressure [mTorr]	40	20	30
Primary source [W]	2500	2500	2500
Secondary source [W]	0	400	400
Platen power [W]	0	50	50

**Gas flows [sccm]**			
SF_6_	1	120	400
C_4_F_8_	300	1	1
O_2_	1	120	1

### 4.3 Device design

The geometry of the shadow mask used for this proof-of-principle study, featuring a 1 mm × 7 μm bridge, is designed to form two-terminal devices where exfoliated flakes cover the predefined contact gaps. The contact dimensions are optimized for the typical size and density of exfoliated flakes while ensuring compatibility with the spatial resolution of the micro-beam ARPES setup. A gap size of 4–8 μm is found to provide a balance between the ability to resolve the area between contact pads and maintaining a high probability of flakes bridging. The vertical gap length (1 mm) determines the average amount of flakes covering the gap—longer gaps increase the probability of multiple flakes, while shorter ones are preferred for single-flake transport measurements (at the risk of having no flake bridging the gap). The large lateral extent of the contact pads to the sides (>1 mm) ensures sufficient exposed area next to the tape loop for the fixation of wires to establish electrical contact to the device.

### 4.4 Contact deposition

Ti/Au contacts are deposited on the substrate using the shadow mask in an electron beam evaporator (BAK Uni, Evatec) at pressures below 1 × 10^−6^ mbar. A 2 nm Ti layer is deposited first to promote adhesion between SiO_2_ and the 13 nm Au film.^66^ The thickness of the Au layer is optimized to balance surface smoothness for exfoliation and mechanical durability during electrical pulsing.

### 4.5 Substrates

The devices are fabricated on 4-inch Si/SiO_2_ wafers (MicroChemicals) with a thickness of 525 ± 20 μm, p-type doping, a resistivity of 1–5 mΩ cm, bow/warp <30 μm, and 290 nm of SiO_2_.

### 4.6 Material transfer

After contact deposition, bulk 1T-TaS_2_ crystals with typical dimensions of 1–3 mm in diameter and ≈100 μm thickness are cleaved on tape loops, brought into contact with the substrate—aligning the bulk material by eye with the pre-patterned contacts—and gently pressed on to avoid trapping air pockets under the tape. Among other commonly used tapes, *e.g.* Nitto Blue Tape or Scotch Tape, we achieved the most reproducible results with silicone adhesive, UHV compatible Kapton tape. The exfoliation process strongly depends on the cleanliness and smoothness of the Au surface as contamination and roughness diminish the adhesion between the vdW material and Au, leading to a lower exfoliation yield.^24^ This occurs because airborne organic contaminants accumulate on the Au surface, turning it more hydrophobic and weakening the adhesion.^27^ To minimize these effects, the transfer is performed rapidly after venting the evaporation chamber with N_2_. Material transfer within 5 min consistently yields large-area flakes down to the monolayer limit, whereas longer time spans result in predominantly thicker (≈50–100 nm) flakes with lower yield.

### 4.7 Device assembly

The Si wafer containing 24 devices is cleaved manually into dies and glued on standard flag-style copper plates (see Fig. S1) using Ag epoxy (EPO-TEK H20E, Epoxy Technology). Electrical connections to the device are also made using Ag epoxy by attaching insulated copper wires to the Au pads. The curing of the epoxy is performed under conditions that minimize thermal stress: we heat the fully assembled sample holder to the lowest temperature specified that ensures full curing of the epoxy, *i.e.* 80 °C, thereby avoiding thermal expansion that could strain the vdW flake or introduce residues from melting the adhesive material of the tape.

### 4.8 Electrical measurements

Measurements are carried out using a pulsed current source (Keithley 6221, Tektronix) and nanovoltmeter (Keithley 2182). Resistance values are obtained from a linear fit to the slope of *IV* curves on a ±10 μA current interval. Pulsed *IV* curves to measure the switching to the hidden state are recorded using a pulsed sweep mode, applying current pulses from 0 to 16 mA in 100 μA steps. Each pulse has a width of 200 μs, but the voltage is measured only during the second half of the pulse, *i.e.* after a 100 μs source delay. Between pulses, a pause of 100 ms minimizes heating.

### 4.9 Angle-resolved photoemission spectroscopy

Measurements are conducted at the Spectromicroscopy endstation at Elettra Sincrotrone Trieste with a photon energy of 74 eV and an energy resolution of ≈45 meV. A beam footprint of 1.0 × 1.5 μm^2^ is achieved through focusing with a zone plate and a Schwarzschild objective. Spectra are acquired using a hemispherical analyzer (MB Scientific AB A-1) equipped with a 2D deflector system, accepting a 35° cone. Devices are cleaved at room temperature in a preparation chamber (<1 × 10^−9^ mbar pressures) and transferred to the manipulator head in the main chamber (<7 × 10^−10^ mbar). The cooling rate is controlled at ≈(1–2) K min^−1^ to cross the NCCDW-CCDW transition of 1T-TaS_2_ in a controlled manner. The binding energy axis is aligned to *E*_F_ by fitting the angle-integrated spectrum with a Fermi–Dirac distribution.

### 4.10 Core-level analysis

To analyze the Ta 4f_7/2_ core-level spectra, we employ a multi-step fitting procedure. First, we perform spatial clustering using a *k*-means algorithm^62^ to group different regions of interest based on spectral similarity. Within each cluster, all angle-integrated spectra are averaged to obtain the cluster mean and standard deviation. A Shirley-type background^63^ is subtracted to account for inelastic scattering contributions. The core-level spectra are fitted using three Voigt profiles, corresponding to the three in-equivalent Ta sites of the star-of-David pattern forming the CDW state. The Voigt function, defined as the convolution of a Lorentzian and a Gaussian profile, accounts for both the intrinsic lifetime broadening of the core-hole state (Lorentzian) and the instrumental resolution of the measurement setup (Gaussian). The initial peak positions were set to −23.64 eV, −23.10 eV, and −22.97 eV, based on prior studies on bulk crystals,^64^ and allowed to vary within ±0.2 eV. The initial amplitude ratios were set to 1 : 6 : 6 (A : B : C), and the Gaussian and Lorentzian widths constrained to *σ* ≤ 0.2 and *γ* ≤ 0.2, respectively. The best fit parameters were determined using least-squares optimization. Intensity ratios were established by integrating the peak areas.

### 4.11 Atomic force microscopy

Measurements are conducted after the ARPES experiment using a Dimension 3100 system (Bruker) to establish the thickness and homogeneity of the exfoliated 1T-TaS_2_ flakes. Line profiles were extracted across the flake edges from full-area scans. The resulting profiles (see Fig. S2), were leveled by subtracting the local slope and fitted to determine the step height, which corresponds to the flake thickness.

### 4.12 Scanning electron microscopy

High-resolution images of the shadow mask were obtained using a scanning electron microscope (Regulus 8230, Hitachi).

## Author contributions

N. T., C. B., R. V. and I. G. conceived the project with input from D. K., Y. E., D. M. and S. G. N. T., C. B. and R. V. fabricated the devices. I. G. fabricated the shadow mask with input from D. K. N. T., C. B., R. V., E. D. V., A. M., Y. V., J. L. and A. B. carried out the ARPES experiments. N. T. analyzed the ARPES data with input from S. A. E. N. T., C. B., R. V., I. G., D. M. and S. G. wrote the manuscript with input from all co-authors.

## Conflicts of interest

The authors declare no competing interest.

## Supplementary Material

NR-017-D5NR02125A-s001

## Data Availability

Data of this article, including ARPES and AFM scans, transport data and photographs are available at the Zenodo repository under https://doi.org/10.5281/zenodo.15482370. The Supplementary Information file contains six figures: Details of a fully assembled device on an Omicron-type sample holder (Fig. S1), AFM characterisation of the exfoliated flake discussed in the main article (Fig. S2), a zoom-in of the ARPES spectrum near the Fermi level (Fig. S3) and details of the saptial clustering analysis, including the ‘elbow curve’ (Fig. S4), overlaid optical and core-level based mapping (Fig. S5), as well as background subtraction (Fig. S6). See DOI: https://doi.org/10.1039/d5nr02125a.
