# Supplementary material for: van der Waals devices for surface-sensitive experiments
Source: Nanoscale. 2025 Aug 14;17(34):19957–65. doi: 10.1039/d5nr02125a (PMC12363651; doi:10.1039/d5nr02125a)
Supplement: NR-017-D5NR02125A-s001 [file NR-017-D5NR02125A-s001.pdf]

Supplementary Information (SI) for Nanoscale.
This journal is © The Royal Society of Chemistry 2025

Supplementary Information

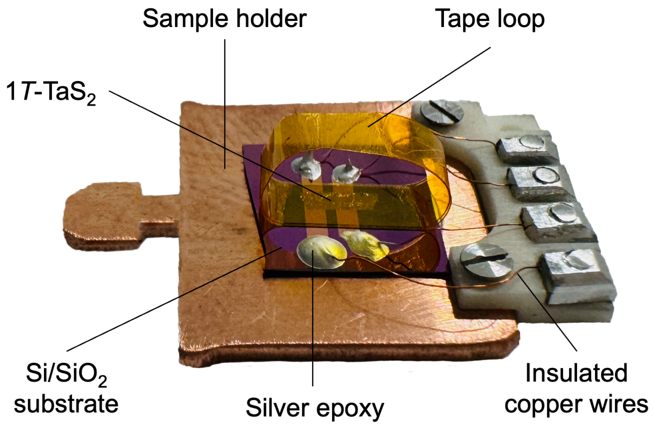

Fig. S1. Fully assembled vdW device on an Omicron-type Cu sample holder. A stencil mask design accommodating two two-terminal devices on a single holder is used here (different from the stencil mask shown in the main text).

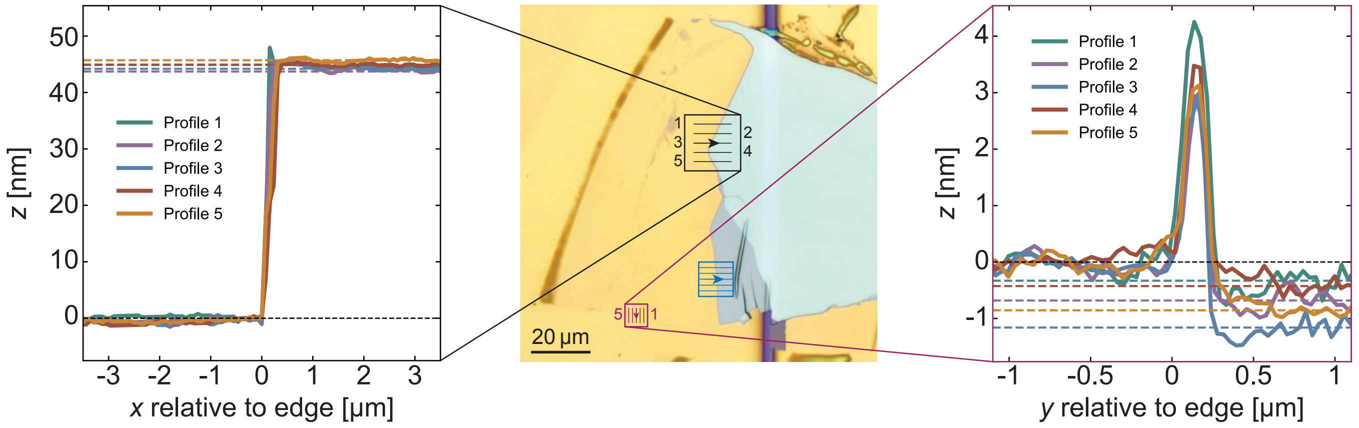

Fig. S2. Representative profiles from AFM measurements taken along the colored boxes with arrows indicating the scan direction. The thickness of the bulk flake region (black box) is measured to be 44.7 ± 0.7 nm, corresponding to ≈ 70 layers of $1T$–TaS_2. The nearly transparent area to the left of the flake (pink box) has a measured thickness of 0.7 ± 0.3 nm and is identified as a monolayer, in reasonably good agreement with the c-axis spacing of bulk $1T$–TaS_2 [59]. The bottom-left corner of the flake (blue box, scans not shown) has a thickness of 23.7 ± 0.9 nm, corresponding to ≈ 40 layers. A prominent peak observed in the monolayer scan just before the step to the substrate is attributed to tape residue at the flake edge from the exfoliation process, which locally affects the AFM signal. The x-direction corresponds to the horizontal scan axis, y is vertical within the sample plane, and z denotes the out-of-plane direction (height) measured by the AFM.

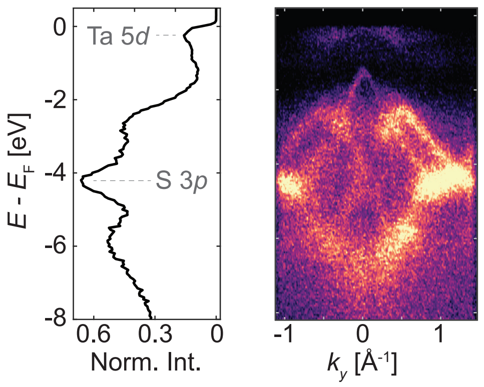

Fig. S3. Zoom-in close to E_F of the ARPES spectrum shown in Fig. 3 of the main text.

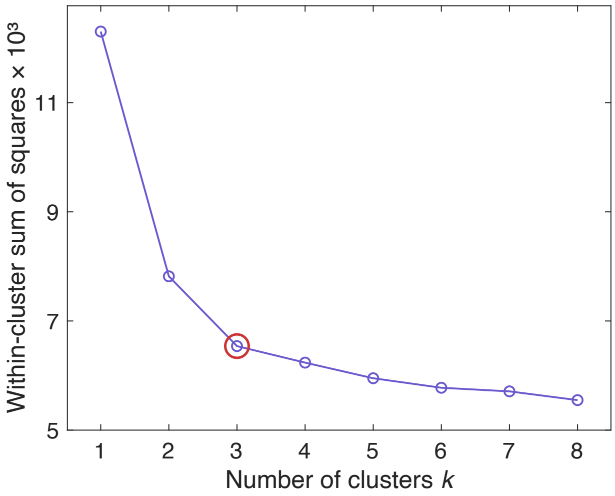

Fig. S4. Within-cluster sum of squares vs. number of clusters k, obtained with the k-means algorithm. The optimal cluster number, here $k = 3$, is determined using the "elbow" method, where the sum of squares exhibits diminishing returns.

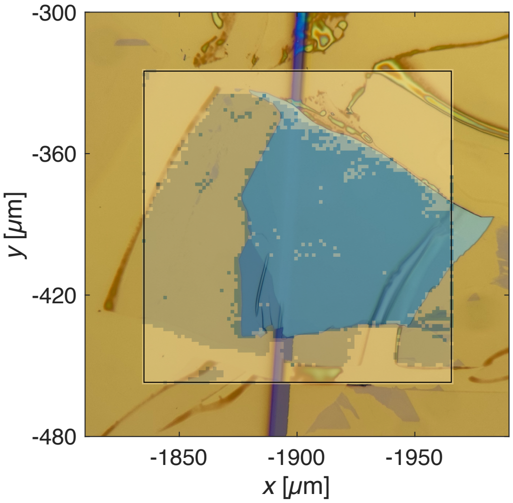

Fig. S5. Spatial distribution of clusters, obtained from k-means clustering of the Ta $4f$ core-level spatial mapping, overlaid on an optical microscope image. The clustering based on the spectral signatures overlaps well with three distinct regions, namely $1T$–TaS_2 with bulk-like (blue) and monolayer (gray) properties, as well as the gold surface (yellow).

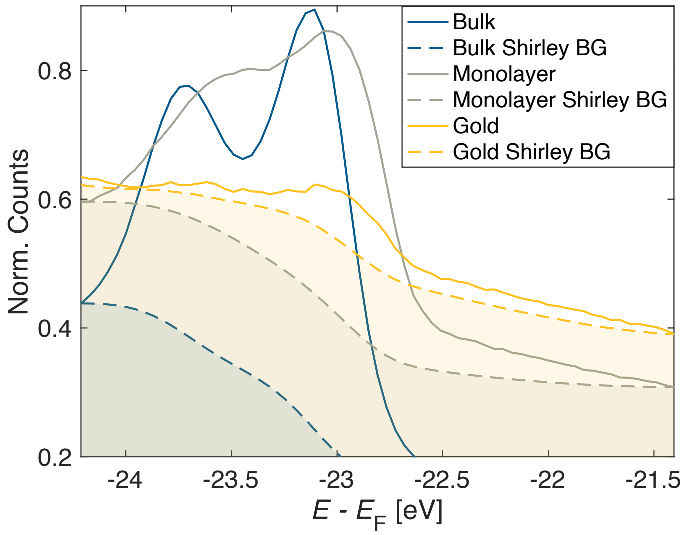

Fig. S6. Averaged angle-integrated spectra within a cluster representing distinct regions of the $1T$–TaS_2 device, *i.e.* bulk and monolayer flake, as well as gold surface (solid lines). The curves are fitted with three Voigt profiles, in addition to accounting for a Shirley-type background (BG, dashed lines).
